# The efficiency of varying methods and degrees of time compensation for the solar azimuth

**DOI:** 10.1098/rsbl.2023.0355

**Published:** 2023-11-22

**Authors:** Richard Massy, Karl R. Wotton

**Affiliations:** Centre for Ecology and Conservation, University of Exeter, Cornwall Campus, Penryn, UK

**Keywords:** migration, orientation, time-compensated sun compass, navigation

## Abstract

Daytime migrants are known to orientate using the position of the sun, compensating for its changing position throughout the day with a ‘time-compensated sun compass'. This compass has been demonstrated in many migratory species, with various degrees of accuracy for the actual movement of the sun. Here, we present a model for differing levels of compensation for the solar ephemeris that shows that a high degree of efficiency, in terms of distance travelled, can be achieved without full time compensation. In our model, compensating for the sun's position had a diminishing return with an accuracy of 80% leading to only a 2% reduction in distance travelled. We compare various modes of time compensation—full, partial, time averaged and step—revealing their directional efficiency in terms of distance travelled under an autumn migration scenario. We find that the benefit of time compensation varies with latitude, with time averaging performing very well, especially at all high latitudes, but step compensation performing better at very low latitudes. Importantly, even rudimentary adjustment can dramatically increase the efficiency of migration, which suggests an easy pathway for the independent evolution of time compensation.

## 1. Introduction

Diurnal animals can use the sun to orientate, and while many use it just to detect changes in heading, others use it as a compass to provide absolute direction [1]. The use of the sun as a compass is important for migrating animals as they need to travel long distances in a specific direction, and it likely forms the primary cue for many day flying migrants [2,3]. Using the sun effectively at different times of day however requires compensating for its shifting position, which is known as a ‘time-compensated sun compass', and has been identified in birds [2], in insects such as the monarch butterfly (*Danaus plexippus*) [4], the neotropical butterflies *Aphrissa statira* and *Phoebis argante* [5] and the marmalade, vagrant, pied and yellow-clubbed hoverflies (*Episyrphus balteatus, Eupeodes corollae, Scaeva pyrastri* and *Scaeva selenitica*) [3,6], and in Atlantic herring *Clupea harengus* [7].

To compensate for the changing position of the sun, migrants must have an internal representation of the sun's movement. Three factors affect the rate of change of the sun's azimuth: time of day, date and latitude, and the interactions between these factors mean that converting azimuth into an exact bearing is a complex operation that may not be strictly necessary for efficient migration. To simplify this calculation, one suggested method, known as time averaging, assumes the azimuth changes at a constant rate throughout the day [5]. Seasonal variation can be accounted for by adjusting the rate of compensation by the number of daylight hours, as observed in crustacean sandhoppers [8]. An alternative method is the step function, where the sun is assumed to be in the east in the morning, with an abrupt adjustment at midday to the west, as shown in honeybees [9], although is unlikely to be used by migrating butterflies [10]. The sun compass may also be only partially time-compensated, and this has been discussed in the literature by several authors [1,5,11]. Partial compensation has been seen in several groups (sandhoppers [12], honeybees [13] and pigeons [14–16]). For example, desert ants underestimate the rate of movement of the sun's azimuth when it is high and overestimate this rate of movement when it is low [17]. Here, we quantify migratory efficiency, in terms of distance travelled, under varying levels and mechanisms of time compensation.

## 2. Methods

### (a) Estimating the sun's azimuth for separate locations

Sun azimuth data from September, a peak month for autumn migration, were obtained for four known migration locations of different latitudes through which a diverse range of bird and insects migrate (the data year [2018] does not affect results). The highest latitude was the Falsterbo peninsula, Sweden (55.36° N, 12.81° E) [18–20], followed by the Pyrenean mountain pass of Bujaruelo (42.70° N, 0.06° W) [21,22], the Maghreb, Morocco (31.46° N, 7.83° W) [23–26], and lakes in Panama (9.17° N, 79.85° W) [5,10]. The data were downloaded from sunearthtools.com at 5 min intervals, so to estimate the sun's azimuth for the intermediate minutes, spline models were fitted to daily azimuth data using the smooth.spline(…, all.knots=T) and predict() functions from base R version 4.2.1.

### (b) Full compensation

To simulate the efficiency of a time-compensated sun compass, which only partially accounts for the changing solar ephemeris, the sun azimuth data were used to simulate 100 levels of time compensation ranging from 0% to 100%. The directional efficiency of each compensation level was determined for every daylight minute of the month of September 2018 and averaged over the entire season.

The simulations are based on a southward migration, travelling towards the sun, with the assumption that organisms travelling in different directions also undergo menotaxis to orientate to their desired direction. The null model of time compensation is phototaxis, with the direction of travel being the sun's azimuth, with full compensation being a perfectly direct flight. Partial levels of compensation are when intermediate adjustments are made, with the compensation level referring to the proportion of the adjustment made for full compensation. For example, with an azimuth (*α*_min_) of 145°, the full compensation adjustment for the desired migration direction of 180° is +35°, so a 60% level of compensation (c = 0.6) would correspond to an adjustment of +21° and a heading of 166°.

$$\mathrm{directional}\;{\mathrm{efficiency}}_{c\alpha \;\min}=\;-\cos(\alpha_{\min}+c(180-\alpha_{\min}))$$

### (c) Time averaging

To simulate a time-averaging approach to sun compensation, the directional efficiency of a wide range of azimuth adjustment rates ranging from 0° to 40° per hour was calculated, with adjustments made relative to the time of the solar noon. For example, on a day when solar noon was at 13.56 (*t*_zenith_), using an angle adjustment rate (s) of 15° per hour means that at 11.56 (*t*_min_), 2 h before noon, the angle adjustment would be +30°. The sun's azimuth (*α*_min_) of 145° would result in a flight vector of 175°.

$$\mathrm{directional}\;\;{\mathrm{efficiency}}_{sa\;\min}=\;-\cos(\alpha_{\min}+s(t_{\mathrm{noon}}-t_{\min}))$$

### (d) Step compensation

To simulate step compensation a single adjustment, (*s*), was added or subtracted depending upon if the time (*t*_min_) was before or after the solar noon (*t*_noon_).

$$\begin{array}{rl} & \mathrm{directional}\;\;{\mathrm{efficiency}}_{sa\;\min\;(t\min\;<\;t\mathrm{noon})}=\;-\cos(\alpha_{\min}+s)\\ & \mathrm{directional}\;\;{\mathrm{efficiency}}_{sa\;\min\;(t\min\;>\;t\mathrm{noon})}=\;-\cos(\alpha_{\min}-s) \end{array}$$

### (e) Weighting

Migratory activity is not constant and can vary between taxa from all day, to tailing off around sunrise and sunset, to concentrated at a particular daylight hours [21,27–29]. To show how migratory efficiencies change in organisms where migration peaks at midday, as seen in many insects and some birds [27,28], we used radar data of hoverflies migrating in the UK in September [29]. A spline fitted to this daily abundance data (electronic supplementary material, figure S2) was consulted to weight each minute (*w*_min_) of the experimental period according to the proportion of individuals migrating.

$$\mathrm{directional}\;\;\mathrm{efficiency}=\overset{N_{\min}}{\underset{\min=1}{\sum }}\mathrm{directional}\;\;{\mathrm{efficiency}}_{\min}*\;\frac{w_{\min}}{\sum w_{N\min}}\;$$

## 3. Results

**Figure 1.**
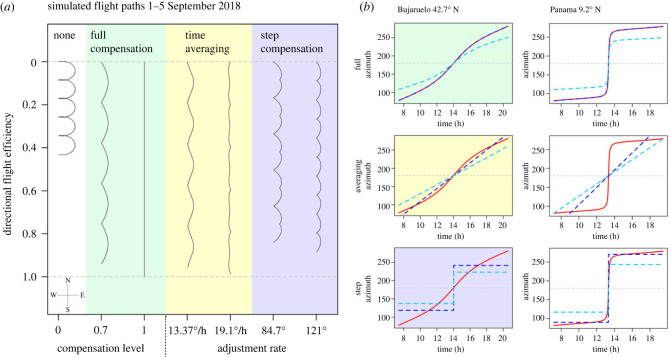
(*a*) Virtual flight paths of different migration strategies generated from sun position data from the Puerto de Bujaruelo 1–5 September. From left to right: none (white)—phototaxis shows the flight path when orientated towards the sun. Full compensation (green)—compensation relative to the sun's azimuth, with ‘compensation level' referring to the degree of compensation. Time averaging (yellow)—adjusting at a constant rate throughout the day. Step compensation (purple)—a single large direction adjustment at midday. (*b*) Real and estimated sun azimuths based upon different compensation strategies at the Puerto de Bujaruelo (left) and Panama (right). From top to bottom: full, time averaging, step. Red lines: real sun azimuths; dark blue lines: 100% of the optimum level of compensation; light blue: 70%; grey: no compensation/phototaxis.

**Figure 2.**
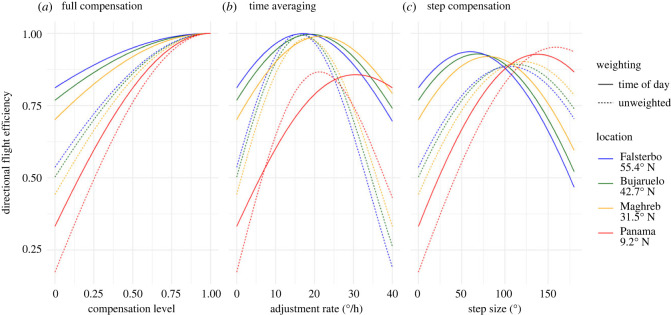
The efficiency of simulated time-compensated sun compass strategies relative to a direct flight. Solid lines are weighted by the temporal abundance of migrants; dotted lines are unweighted. Colours correspond to sun azimuth data from locations of differing latitudes. (*a*) Full compensation adjusts relative to the position of the sun and the desired direction of migration, ranging from compensation level 0 (phototaxis, flying in the direction of the sun's azimuth) to 1 (perfect time compensation, a direct flight). (*b*) Time averaging is azimuth compensation at a constant rate throughout the day relative to the time of solar noon. (*c*) Step compensation is a large single adjustment for sun position at midday.

To model the effectiveness of imperfect versus perfect time compensation, we conducted simulations of autumnal southward migrations under six conditions: three time-compensation strategies (full compensation, time averaging and step compensation) under two differing weighting criteria (by time of day and unweighted; a comparison of weighting methods is made in electronic supplementary material, figure S6). Flight directedness affected the directional efficiency equally for all levels of compensation in proportion to the degree of directedness and so was not considered further (electronic supplementary material ‘Modelling inaccuracy'). Simulated migration tracks and estimated sun azimuths are shown in figure 1 while results are visualized in figure 2.

### (a) Full compensation

The efficiency of phototaxis varied greatly between locations from 17% to 54% of a perfectly direct flight; this increased to 33% to 81% when weighted by time of day (figure 2*a*). Both weighted and unweighted simulations converge to 100% at full compensation so the benefit of time compensation was higher when unweighted for time of day. Full time compensation showed diminishing returns, with 70% time compensation, similar to the observed level in Massy *et al.* [3], providing 90% of the increase in efficiency of complete compensation (figure 2*a* and table 1).

**Table 1.** Varying levels of time compensation with extrapolated distances travelled in kilometres per day at 10 m s^−1^ (medium insect [6]) or 16 m s^−1^ (average migratory bird [30]) for two locations, assuming 4 h of flight. Bracketed values are weighted by time of day.

level of time compensation	Puerto de Bujaruelo (42.70° N)			Lake Gatun, Panama (9.17° N)		
	directional flight efficiency	km per day at 10 m s^−1^	km per day at 16 m s^−1^	directional flight efficiency	km per day at 10 m s^−1^	km per day at 16 m s^−1^
PHOTOTAXIS	0.503 (0.768)	72 (111)	116 (177)	0.174 (0.332)	25 (48)	40 (76)
0.2	0.664 (0.847)	96 (122)	153 (195)	0.434 (0.546)	62 (79)	100 (126)
0.4	0.803 (0.912)	116 (131)	185 (210)	0.664 (0.733)	96 (106)	153 (169)
0.6	0.910 (0.960)	131 (138)	210 (221)	0.845 (0.877)	122 (126)	195 (202)
0.8	0.977 (0.990)	141 (143)	225 (228)	0.960 (0.969)	138 (139)	221 (223)
FULL	1 (1)	144 (144)	230 (230)	1 (1)	144 (144)	230 (230)

### (b) Time averaging

The directional efficiency of different adjustment rates of time averaging followed a parabolic curve. The optimum adjustment rate decreased, and the peak efficiency increased with latitude (table 2 and figure 2). Weighted simulations had higher optimum adjustment rates, reflecting that weighting favours the middle of the day when the sun's azimuth moves more quickly. Similarly, using the Puerto de Bujaruelo as an example, the optimum adjustment rate reduced throughout the month of September from 20.4 _±0.1_° h^−1^ to 17.8 _±0.1_° h^−1^, reflecting the sun's azimuth movement rate slowing from a mean of 15.5° h^−1^ to 14.7° h^−1^ (see electronic supplementary material, figure S6). Using the optimum adjustment rate for each day further increased the potential efficiency from 0.9969 to 0.9974 for weighted simulations and from 0.9938 to 0.9941 for unweighted simulations.

**Table 2.** The optimum levels of different time-compensation strategies at varying geographical locations. Peak efficiencies are relative to a perfectly direct flight. Bracketed values are weighted by time of day.

	Falsterbo, Sweden	Puerto de Bujaruelo, France	Maghreb, Morocco	Neotropical, Panama
latitude	55.36°	42.70°	31.46°	9.17°
phototaxis efficiency	0.537 (0.812)	0.503 (0.768)	0.442 (0.701)	0.174 (0.332)
full compensation: increase over phototaxis	86% (23%)	99% (30%)	126% (43%)	475% (201%)
time averaging: optimum hourly adjustment	16.1° (17.1°)	17.0° (19.0°)	18.1° (21.5°)	21.3° (30.0°)
time averaging: peak efficiency	0.998 (0.999)	0.994 (0.997)	0.983 (0.990)	0.867 (0.857)
time averaging: increase over phototaxis	86% (23%)	98% (30%)	122% (41%)	398% (158%)
step compensation: optimum adjustment	105° (60°)	112° (68°)	121° (81°)	159° (138°)
step compensation: peak efficiency	0.886 (0.937)	0.893 (0.929)	0.902 (0.920)	0.952 (0.928)
step compensation: increase over phototaxis	65% (15%)	78% (21%)	104% (31%)	447% (180%)

### (c) Step compensation

Step compensation followed a similar pattern to time averaging, maintaining relatively high peak efficiencies of 95% and 92% for weighted and unweighted simulations at the Puerto de Bujaruelo. The optimum step size fell more dramatically throughout September from 99° to 79° for unweighted simulations. In contrast to time averaging, the benefit of step compensation was higher at low latitudes due to closely matching the sun's azimuth, with a higher directional efficiency than time averaging in Panama (9.2° N) (figure 1 and table 2).

## 4. Discussion

Our results show that time compensation vastly increases the efficiency of sun-compass-based migration. Due to the parabolic curve of the efficiency of all time compensation strategies, modest levels of time compensation provide most of the benefit. When simulating a southward migration during the month of September, we found only a 5% loss in efficiency between complete time compensation and a partial time compensation of 70% (as seen in *Scaeva* spp*.* hoverflies [3] and in neotropical butterflies [5,31]).

The sun's azimuth follows a sigmoidal curve with respect to time, which time averaging simplifies by linearization [5,11]. At temperate latitudes even this linear adjustment resulted in barely any loss of directional efficiency compared to full compensation. The predicted azimuth of step compensation deviated from the sun's azimuth more than time averaging, resulting in lower efficiency, although the deviation was only sufficient at certain times of day—dawn, midday and dusk—to produce a palpable decrease. The pattern reversed at low latitudes where step compensation performed better than time averaging because the z-shape of the predicted azimuth of step compensation more closely resembled the path of the sun's azimuth than the straight line of time averaging. The (co)sinusoidal relationship between vector and directional efficiency means that small deviations from the intended direction have a miniscule impact. The opposite is true for horizontal drift, so it is likely that goal-orientated migrants like monarch butterflies require more directional accuracy to reach their destination (reflected in high directedness in simulation experiments [4,32]). Since drift produced by time compensation is symmetrical throughout the day, this is unlikely to influence the choice of time-compensation strategy, however.

Latitude greatly influenced the efficiency of migration only by phototaxis, which was higher in northerly latitudes where the sun's path was more southerly. The potential benefit of time compensation correspondingly ranges from doubling the efficiency of migration in Sweden, to a sixfold increase in Panama. The increased benefit close to the equator is balanced by increased complexity as the sun's azimuth moves further, faster and with differing speed and is further exacerbated by high solar elevations reducing orientational efficiency, as shown in dung beetles [33]. The simple step function fit the path of the sun very well, but butterflies migrating through Panama appear not to use it [10], perhaps because an effective strategy also needs flexibility to work in other latitudes and times of year. While time averaging performed poorly here compared to more northerly latitudes, it still represented a fivefold increase in efficiency that was only marginally improved upon by the sixfold increase of full compensation. Ultimately the compensation strategy does not have to be linear, and even adjusting for the speed of the sun in a rudimentary way, as shown in desert ants [17], would provide a benefit almost indistinguishable from full compensation.

To estimate distance travelled we use the examples of migratory hoverflies and moths in western Europe, which had autumn displacement speeds of around 10–13 m s^−1^ recorded by radar [6]. At 10 m s^−1^, a hypothetical migration of 1500 km from southern England to the south of Spain of would take 14 days if using only phototaxis and optimizing 4 h of flight around the middle of the day (i.e. weighted). Time-compensating for sun position at a level of 70% would reduce this to 11 days representing an advantage that time compensating fully would barely improve upon. The directional efficiency of autumnal migration is likely to be under strong selection as it occurs during a period of deteriorating weather conditions when the cost of failure is high. Intermittently suitable weather conditions might mandate migrating whenever it is favourable, including the mornings and evenings when compensation is more valuable as the sun is in a less southerly position. In this case (migrating equally all day: unweighted), 70% compensation still allows this migration to be undertaken in 11 days, whereas via phototaxis it would take 21 days.

The sun is the most reliable directional cue, which with menotaxis allows many animals to travel in their desired direction [34]. Time compensation vastly increases the efficacy of sun-based navigation, with even rudimentary compensation methods providing sizable increases to the efficiency of travel and is therefore likely to be under strong selection. In addition, a wide variety of organisms, from birds to insects to mammals, use a time-compensated sun compass to orientate suggesting that it has independently evolved on many occasions. The manner in which the sun is used differs: while many organisms appear to use the sun as their primary cue during long-distance movements, there may be significant variation in how it is integrated with other senses such as wind, olfaction, the magnetic compass or the use of landmarks [35–39]. With such diverse senses, neural architecture and life histories, and the fact that the optimal strategies are dependent on location, it is likely that sun azimuth change has been approximated in different ways and to different extents. While individual requirements, such as goal-oriented migration, might mandate more accuracy, this study shows that it is possible to efficiently navigate with approximations of sun position and partial levels of compensation.

## Data Availability

Supplementary material is available online [40].
